# PP1-associated responses linked to HMGR-related changes during postharvest squalene accumulation in *Camellia oleifera* seeds

**DOI:** 10.3389/fpls.2026.1895304

**Published:** 2026-08-12

**Authors:** Jianwen Wu, Jihua Guan, Mi Qiu, Wen Chen, Guiqing Li

**Affiliations:** Guangxi Forestry Research Institute, Guangxi Laboratory of Forestry, Nanning, China

**Keywords:** 3-Hydroxy-3-methylglutaryl-CoA reductase, *camellia oleifera*, high-temperature and high-humidity treatment, mevalonate pathway, protein phosphatase 1, squalene

## Abstract

Plants synthesize diverse isoprenoids that are essential for growth, development, and adaptation to environmental stress. In *Camellia oleifera*, squalene is a valuable minor constituent of seed oil, yet the regulatory processes linking postharvest stress to squalene accumulation remain poorly understood. Here, we investigated the relationship between protein phosphatase 1-associated responses and changes related to 3-hydroxy-3-methylglutaryl-coenzyme A reductase during postharvest squalene accumulation in *Camellia oleifera* seeds exposed to 35 °C and 95% relative humidity. Squalene content increased rapidly and reached approximately 2.6 times its initial level after 24 h. By contrast, the enzyme-linked immunosorbent assay-based immunoreactive signal associated with 3-hydroxy-3-methylglutaryl-coenzyme A reductase increased more gradually and remained approximately 30–40% above the initial level from 24 to 48 h. Transcriptome analysis showed that *CoHMGR2*, the most abundant 3-hydroxy-3-methylglutaryl-coenzyme A reductase transcript at 0 and 12 h, declined during treatment, whereas several downstream genes in the mevalonate pathway showed treatment-responsive increases. These contrasting patterns indicate that the observed response associated with 3-hydroxy-3-methylglutaryl-coenzyme A reductase could not be explained by transcript abundance alone. Two genes encoding protein phosphatase 1 catalytic subunits also showed increased expression. Recombinant-protein pull-down assays supported an association *in vitro* between a *Camellia oleifera* 3-hydroxy-3-methylglutaryl-coenzyme A reductase protein and a protein phosphatase 1 catalytic subunit under the defined assay conditions. Metabolomic profiling revealed extensive metabolic reorganization during the major phase of squalene accumulation. Pull-down proteomic analysis further identified candidate proteins associated with 3-hydroxy-3-methylglutaryl-coenzyme A reductase that were functionally annotated to mitochondrial energy metabolism, redox homeostasis, protein folding, and membrane trafficking. Collectively, these findings support a cautious model in which protein phosphatase 1-associated responses are linked to changes related to 3-hydroxy-3-methylglutaryl-coenzyme A reductase and to reorganization of the mevalonate pathway during postharvest squalene accumulation. Whether protein phosphatase 1 directly regulates 3-hydroxy-3-methylglutaryl-coenzyme A reductase through dephosphorylation remains to be determined by targeted biochemical and phosphoproteomic analyses. This study extends the current protein phosphatase 2A-centered framework of plant 3-hydroxy-3-methylglutaryl-coenzyme A reductase regulation and supports protein phosphatase 1 as a candidate component of a regulatory context associated with sterol and triterpene metabolism in a woody oil crop.

## 1 Introduction

*Camellia oleifera* Abel. (*C. oleifera*) is a traditional woody oil crop extensively cultivated in southern China, where its planting area now exceeds 5 million ha and its seed oil constitutes a major regional edible-oil resource. The oil is characterized by a high proportion of unsaturated fatty acids (>85%), dominated by oleic acid (76–81%), which confers a “Mediterranean-like” fatty-acid profile and has led to its designation as “oriental olive oil.” In addition to triacylglycerols, *C. oleifera* oil contains minor bioactive constituents, including squalene, phytosterols, tocopherols, polyphenols, and triterpenoid saponins, which together are associated with antioxidant, anti-inflammatory, lipid-lowering, and cardioprotective effects. Notably, *C. oleifera* oil typically contains approximately 122–248 mg/kg squalene (Wang et al., 2017), placing *C. oleifera* among the richer plant sources of this triterpenoid. However, previous studies of squalene and triterpenoid metabolism in *C. oleifera* have focused primarily on seed development and transcriptional regulation. These studies identified *CoHMGR2* as a member of the 3-hydroxy-3-methylglutaryl-CoA reductase gene family whose expression is closely associated with squalene accumulation, *CoSQS* as the gene encoding squalene synthase, and *CoWRKY15* as a WRKY transcription factor that activates *CoSQS* expression. Collectively, these findings have advanced our understanding of developmental and transcriptional control of squalene biosynthesis in *C. oleifera*. Nevertheless, whether defined postharvest treatments can promote squalene accumulation, and whether this response involves regulation of HMGR beyond the transcript level, remain largely unknown (Zhang et al., 2022).

In plants, the cytosolic mevalonate (MVA) pathway supplies precursors for a diverse array of isoprenoid products, including sterols involved in membrane biogenesis, sesquiterpenoid phytoalexins and steroidal glycoalkaloids involved in defense, brassinosteroids and cytokinins involved in growth regulation, and prenyl groups, dolichols, and ubiquinone required for protein modification and cellular respiration (Stermer et al., 1994; Lange et al., 2015; Rodríguez-Concepción and Boronat, 2015; Kopcsayová and Vranová, 2019). 3-Hydroxy-3-methylglutaryl-CoA reductase (HMGR) catalyzes the NADPH-dependent reduction of 3-hydroxy-3-methylglutaryl-CoA to mevalonate and is generally regarded as a major rate-controlling enzyme of the MVA pathway. In plants, HMGR is generally encoded by a small multigene family whose members frequently exhibit distinct spatial, developmental, and stress-responsive expression patterns. In *Arabidopsis thaliana* (*A. thaliana*), two genes, *HMG1* and *HMG2*, encode three HMGR protein isoforms: HMGR1S functions as a housekeeping enzyme, whereas HMGR1L and HMGR2 show more restricted expression patterns (Lumbreras et al., 1995). In *C. oleifera*, the HMGR family is expanded: four CoHMGR genes display expression patterns that are tightly correlated with squalene accumulation in developing seeds, with *CoHMGR2* showing the strongest association (Gu et al., 2025). HMGR abundance and activity are regulated at multiple levels. At the transcriptional level, HMGR gene expression responds to phytohormones, Ca²^+^/calmodulin, light, and diverse biotic and abiotic cues. At the post-translational level, HMGR activity is regulated through the N-terminal membrane-spanning region and phosphorylation of a conserved Ser residue by SnRK1-type kinases, which suppresses catalytic activity and constrains mevalonate-pathway flux (Dale et al., 1995). Notably, studies in *A. thaliana* further show that B″-type regulatory subunits of protein phosphatase 2A (PP2A) bind the N-terminal region of HMGR1 isoforms and that PP2A exerts multilevel control by positively influencing *HMG1* transcript abundance while negatively affecting HMGR protein abundance and enzymatic activity (Leivar et al., 2011). To date, PP2A is the only protein phosphatase reported to regulate plant HMGR *in vivo*, leaving open whether additional phosphatases contribute to HMGR control, particularly in woody oil crops.

Reversible phosphorylation on Ser/Thr residues is mediated by a limited set of protein phosphatases that counterbalance a far larger kinase repertoire. Plant Ser/Thr phosphatases are mainly grouped into the PPP family (including PP1, PP2A, PP2B, and PP4–PP7) and the metal-dependent PPM/PP2C family (Barford et al., 1998). Within the PPP family, protein phosphatase 1 (PP1) is a prototypical type-1 serine/threonine phosphatase whose catalytic subunit is highly conserved across eukaryotes. Its functional diversity arises primarily through association with regulatory and targeting proteins that confer subcellular localization and substrate specificity (Takemiya et al., 2009). PP1 substrate specificity is generated largely at the holoenzyme level: regulatory or targeting proteins recruit the broadly active catalytic subunit to defined substrates and subcellular contexts through short linear docking motifs, including RVxF and auxiliary interfaces such as SILK, MyPhoNE, ΦΦ (phi–phi), and SpiDoC (Bollen et al., 2010; Heroes et al., 2013; Peti et al., 2013; Lin et al., 2019). Similar regulatory-subunit-based mechanisms have been documented in plants, where PP1-associated proteins modulate PP1 localization, activity, and stress- or light-related signaling (Takemiya et al., 2013; Zhang et al., 2020). This combinatorial “PP1 toolkit” is estimated to generate hundreds of distinct holoenzymes that localize PP1 to defined subcellular compartments and restrict it to particular substrates (Takemiya et al., 2009). Functional studies in fungi and animals have established PP1 as a central phosphorylation “reset” enzyme that reverses extensive phosphorylation changes during transitions in metabolism, cell-cycle progression, and stress recovery. In plants, pharmacological studies using okadaic acid, calyculin A, and related inhibitors have established PP1/PP2A-type phosphatases as major okadaic acid-sensitive Ser/Thr phosphatases, although inhibitor-based assays generally cannot unambiguously distinguish PP1 from PP2A *in vivo*. More broadly, plant PPP-family phosphatases—including PP1, PP2A, PP4, PP5, PP6, and related subfamilies—are central components of reversible phosphorylation networks and have been implicated in hormone signaling, cytoskeletal regulation, and biotic and abiotic stress responses (Luan, 2003; Wen et al., 2012; Chen et al., 2014; Hu et al., 2014; Durian et al., 2016). However, most mechanistic work has focused on PP2A, including the identification of Bʺ-type PP2A subunits that interact with HMGR and control sterol biosynthesis at multiple levels in *A. thaliana* (Leivar et al., 2011). By contrast, direct substrates of plant PP1 in primary metabolic pathways remain poorly defined and, despite its abundance and central position in stress-signaling networks, no study has yet demonstrated PP1 acting on HMGR or other enzymes catalyzing committed steps in the MVA pathway. This gap leaves open the possibility that a PP1-based phosphatase module could integrate stress cues with isoprenoid-flux control, but such a mechanism has so far escaped experimental attention.

At the molecular level, recent work in *C. oleifera* has largely focused on the regulation of squalene and related triterpenoids during seed development. Comparative transcriptomic and metabolomic analyses have revealed an expanded HMGR gene family, and four *CoHMGR* genes exhibit expression patterns that closely track the dynamic squalene profile in developing seeds, with *CoHMGR2* showing the strongest association with the key accumulation period (Gu et al., 2025). Downstream of the HMGR-catalyzed step, transcriptional control of squalene biosynthesis has also been documented. The WRKY transcription factor *CoWRKY15* binds W-box elements in the *CoSQS* promoter and activates *CoSQS* expression in a light-dependent manner, thereby promoting squalene and triterpenoid biosynthesis in developing seeds (Li et al., 2024). Together, these studies establish developmental stage and nuclear transcriptional regulation as major determinants of squalene production in *C. oleifera*, but they focus almost exclusively on the seed-filling phase.

By contrast, how postharvest handling of fresh seeds modulates squalene accumulation remains largely unknown. Processing-oriented studies have shown that pretreatment, drying/roasting regimes, and extraction strategies markedly influence the composition of *C. oleifera* oil and the retention of unsaponifiable components, indicating that triterpenoid-rich fractions are sensitive to technological conditions (Wang et al., 2017; Zhang et al., 2022). However, systematic analyses of how defined postharvest high-temperature and high-humidity (HTHH) treatments affect squalene biosynthesis at the tissue level are still lacking. At the post-translational level, the only phosphatase demonstrated to directly regulate plant HMGR to date is protein phosphatase 2A (PP2A) in *A. thaliana*, where B″-type PP2A regulatory subunits bind the N-terminal region of HMGR1 isoforms and modulate *HMG1* transcript abundance, HMGR protein stability, and enzymatic activity (Leivar et al., 2011). Whether woody oil crops such as *C. oleifera* harbor analogous or distinct phosphatase–HMGR modules, and whether and how HMGR phosphorylation changes in response to postharvest HTHH stress, remain unresolved.

In this study, we integrated postharvest biochemical measurements, transcriptome-metabolome profiling, and recombinant protein-interaction assays to characterize PP1-linked HMGR responses during HTHH-induced squalene accumulation in *C. oleifera* seeds. We followed the temporal dynamics of squalene accumulation together with an ELISA-based HMGR-related immunoreactive readout, examined the expression patterns of HMGR/MVA-pathway genes and PP1 catalytic-subunit homologs, and assessed the *in vitro* association between a recombinant *CoHMGR* isoform and *CoPP1*. This integrated framework places the *CoHMGR–CoPP1* association within a broader postharvest metabolic and regulatory context and provides a basis for evaluating PP1-associated HMGR regulation in woody oil crops.

## 2 Materials and methods

### 2.1 Plant materials and postharvest HTHH treatments

Fully mature *C. oleifera* fruits were harvested from the same experimental plantation in Guangxi, China, shortly after the Shuangjiang solar term (late October to early November), when the seeds had reached full physiological maturity. The pericarps were removed immediately after harvest, and intact seeds, including the seed coat, were collected for all subsequent analyses. Hereafter, these samples are referred to as seeds. Seeds were incubated in a controlled-environment chamber at 35 °C and 95% relative humidity and sampled at 0, 6, 12, 24, 36, and 48 h after treatment onset. This regime was defined as the postharvest high-temperature and high-humidity treatment used in this study. The 35 °C treatment was selected based on our previous postharvest optimization study and was applied here as a defined, processing-relevant condition for examining early HMGR-associated responses and squalene accumulation (Wu et al., 2020). For each treatment time point, three biological replicates were prepared (n = 3). Each biological replicate consisted of seeds pooled from 20 fruits within the same treatment batch, corresponding to approximately 40 seeds per replicate. This pooled-sampling strategy was used to capture fruit-to-fruit and seed-to-seed variation while providing sufficient material for subsequent biochemical, transcriptomic, proteomic, and metabolomic analyses. Samples were flash-frozen in liquid nitrogen and stored at −80 °C until analysis.

Samples collected at 0, 6, 12, 24, 36, and 48 h were designated T-0, T-6, T-12, T-24, T-36, and T-48, respectively. Samples from all six time points were used for squalene quantification and the HMGR-related ELISA readout. T-0, T-12, and T-24 samples were used for transcriptomic, metabolomic, and RT-qPCR analyses to represent the baseline, rising, and peak phases of squalene accumulation, respectively. T-0 and T-36 samples were used for GST–CoHMGR pull-down assays, immunoblotting, and pull-down proteomic profiling. All quantitative measurements were expressed on a seed fresh-weight basis where applicable.

### 2.2 Determination of squalene content and HMGR-related ELISA readout

Squalene was quantified by gas chromatography–mass spectrometry using a TRACE GC 1300–ISQ LT system equipped with a TG-5MS capillary column (30 m × 0.25 mm, 0.25 μm; Thermo Fisher Scientific). Finely ground seed tissue was extracted twice with dichloromethane by ultrasonication, and the combined extracts were adjusted to 10 mL before analysis. Helium was used as the carrier gas at 0.8 mL min⁻¹, with a split ratio of 40:1 and an injection volume of 2 μL. The oven was held at 80 °C for 2 min, increased to 300 °C at 20 °C min⁻¹, and held for 15 min. Mass spectra were acquired under electron-impact ionization at 70 eV in selected-ion monitoring mode using m/z 81, 217, 368, and 386. Squalene was quantified against an external calibration curve prepared from an authentic standard and expressed as μg g⁻¹ seed fresh weight.

The HMGR-related immunoreactive readout was determined using a commercial double-antibody sandwich enzyme-linked immunosorbent assay kit for plant HMGR (Meimian, Jiangsu Meimian Industrial Co., Ltd., China; catalog no. MM-1929O2; lot no. 20260114) according to the manufacturer’s instructions. The kit has a stated detection range of 1.6–65 IU L⁻¹ and uses a mouse monoclonal capture antibody and a horseradish peroxidase-conjugated rabbit polyclonal detection antibody against plant HMGR. Fresh seed tissue (50 mg) was homogenized in 450 μL phosphate-buffered saline, and the clarified extract was diluted fivefold for analysis. Each biological replicate was analyzed in a single well, and concentrations were calculated from the supplied standard curve and corrected for dilution. Absorbance was measured at 450 nm. Values were normalized to seed fresh weight as follows: HMGR-related ELISA readout (IU g⁻¹ FW) = C × V × DF/m, where C is the concentration derived from the standard curve (IU L⁻¹), V is the extraction volume expressed in liters, DF is the dilution factor, and m is the fresh tissue mass expressed in grams. Because the assay quantifies antibody-recognized HMGR-related material rather than the catalytic conversion of HMG-CoA to mevalonate, the resulting values were interpreted as an HMGR-related immunoreactive readout and used only for relative comparisons among treatment time points.

### 2.3 Transcriptomic and metabolomic profiling during HTHH treatment

Seeds collected at T-0, T-12, and T-24 were used for metabolomic and transcriptomic analyses. For metabolomics, frozen seed samples were ground in liquid nitrogen, extracted according to the standard workflow of MetWare (Wuhan, China), and analyzed using a Nexera X2 ultrahigh-performance liquid chromatograph coupled to a 4500 QTRAP mass spectrometer. Metabolites were separated on an Agilent SB-C18 column (1.8 μm, 2.1 × 100 mm) and detected in positive- and negative-ion modes using multiple reaction monitoring. Metabolite annotation was based on the MetWare MWDB library and the Kyoto Encyclopedia of Genes and Genomes Compound database, with retention times and mass-spectral features verified against authentic standards when available. Peak areas were log_2_-transformed and normalized before analysis. Principal component analysis, hierarchical clustering, and Pearson correlation analysis were performed in R. Differentially accumulated metabolites were defined by a variable importance in projection value ≥ 1.0 and |log_2_(fold change)| ≥ 1.0. Orthogonal partial least-squares discriminant analysis and 200-permutation tests were performed using MetaboAnalystR. Pathway enrichment was assessed by hypergeometric testing, with a false discovery rate < 0.05 considered significant.

For transcriptomics, total RNA was extracted using a TRIzol-based kit (Sangon, Shanghai, China), treated with RNase-free DNase I, and assessed for integrity and purity. Messenger RNA libraries were prepared from 2 μg of high-quality RNA using the VAHTS mRNA-seq v2 Library Prep Kit for Illumina and sequenced on an Illumina HiSeq X Ten platform to generate paired-end reads. After adaptor trimming and quality filtering, clean reads were assembled *de novo* using Trinity, and transcripts shorter than 200 bp were removed. The longest transcript in each cluster was retained as a unigene. Functional annotation was performed by BLAST searches against major protein and domain databases, and open reading frames of unannotated sequences were predicted using TransDecoder. Gene Ontology and Kyoto Encyclopedia of Genes and Genomes annotations were assigned accordingly. Transcript abundance was quantified with Salmon and expressed as transcripts per million. Differentially expressed genes were identified using DESeq2 with |log_2_(fold change)| ≥ 1.0 and *q* < 0.05. Gene Ontology and pathway enrichment analyses were performed using hypergeometric tests with Benjamini–Hochberg correction, and categories with a false discovery rate < 0.05 were considered significant.

### 2.4 RT-qPCR analysis of CoPP1c gene expression

To validate the HTHH-responsive expression patterns of *CoPP1c-1* and *CoPP1c-2*, total RNA from seeds collected at T-0, T-12, and T-24 was subjected to reverse transcription quantitative PCR (RT-qPCR). First-strand cDNA was synthesized from 1500 ng of total RNA using Maxima Reverse Transcriptase (Thermo Scientific), random hexamer primers, dNTPs, 5× reaction buffer, and RiboLock RNase inhibitor according to the manufacturer’s instructions. The reverse-transcription program consisted of 25 °C for 10 min, 50 °C for 30 min, and 85 °C for 5 min. The resulting cDNA was diluted 10-fold prior to qPCR analysis.

RT-qPCR was performed on an ABI 7500 Real-Time PCR System using 2× SGExcel FastSYBR Mixture (Sangon, catalog no. B532955). Each 20 μL reaction contained 10 μL of 2× SYBR mixture, 0.4 μL each of forward and reverse primers (10 μM), 2 μL of diluted cDNA template, and 7.2 μL of RNase-free water. The cycling program consisted of an initial denaturation at 95 °C for 3 min, followed by 45 cycles of 95 °C for 15 s and 60 °C for 45 s. A melting-curve analysis was performed after amplification to verify the specificity of each amplicon. Relative transcript levels were calculated using the 2^−ΔΔCt^ method, with T-0 as the calibrator and *SAND* as the internal reference gene. Primer sequences were as follows: *SAND*-F, 5′-TCCAAGTATCAGGCACACCG-3′; *SAND*-R, 5′-TCAATGACGAAACCCGTGTATC-3′; *CoPP1c-1*-F, 5′-GTGCGATGATGAGTGTTGATGAC-3′; *CoPP1c-1*-R, 5′-TGGAGGCATTCCTGGCTTAG-3′; *CoPP1c-2*-F, 5′-TAATGAACAAAGATGTGAGAACTGC-3′; and *CoPP1c-2*-R, 5′-AACAATACGCCATCCGAGC-3′. Amplicon sizes were 142 bp for *SAND*, 119 bp for *CoPP1c-1*, and 113 bp for *CoPP1c-2*. Relative-expression data are presented as means ± SD from three biological replicates. RT-qPCR normalization and reporting followed the 2^−ΔΔCt^ method and MIQE-oriented guidance (Livak and Schmittgen, 2001; Bustin et al., 2025).

### 2.5 Cloning, expression and purification of recombinant GST–CoHMGR and His–CoPP1 fusion proteins

The coding sequence of a heat-responsive C. oleifera HMGR isoform, designated CoHMGR, was amplified from seed cDNA and cloned in frame into the pGEX-6P-1 vector (GE Healthcare) to generate an N-terminal GST fusion (GST–CoHMGR) with a predicted molecular mass of approximately 78 kDa. The coding sequence of a protein phosphatase 1 catalytic subunit, designated CoPP1, was cloned into the pET22b-next vector to produce a C-terminal His-tagged fusion (His–CoPP1; ~45 kDa). All constructs were verified by Sanger sequencing prior to protein production.

Recombinant GST–CoHMGR and GST alone were expressed in *Escherichia coli* using IPTG-inducible conditions recommended for pGEX-based vectors and purified by glutathione affinity chromatography. His–CoPP1 was expressed in *E. coli* carrying the pET22b-next construct and purified by Ni²^+^-NTA affinity chromatography under native conditions. In each case, eluted fractions containing the target proteins were pooled and dialyzed against storage buffer to remove excess salts and imidazole. The concentration of purified proteins was determined by the Bradford assay using bovine serum albumin as a standard. Coomassie-stained SDS–PAGE showed predominant bands near the expected molecular masses of approximately 78, 45, and 26 kDa for GST–CoHMGR, His–CoPP1, and GST, respectively.

### 2.6 GST pull-down assays with recombinant proteins and seed protein extracts

GST pull-down assays were performed to assess the *in vitro* association between CoHMGR and CoPP1 and to enrich candidate CoHMGR-associated proteins from *C. oleifera* seed extracts. Glutathione MagBeads were equilibrated in binding buffer containing 20 mM Tris–HCl (pH 7.5), 150 mM NaCl, and 1 mM dithiothreitol. GST alone was used as the negative-control bait in all assays. For recombinant-protein pull-downs, 200 μg of GST–CoHMGR or GST was immobilized on 30 μL of beads for 2 h, followed by incubation with 500 μg of His–CoPP1 overnight at 4 °C. Beads were washed with binding buffer containing 0.1% NP-40, and bound proteins were eluted in 2× SDS sample buffer. Input, flow-through, and pull-down fractions were analyzed by SDS–PAGE and immunoblotting. For seed-extract pull-downs, soluble proteins were extracted from T-0 and T-36 seeds in buffer containing 50 mM Tris–HCl (pH 7.5), 150 mM NaCl, 1 mM ethylenediaminetetraacetic acid, 1 mM dithiothreitol, 10% glycerol, and protease inhibitors. After centrifugation, 500–1000 μg of clarified seed protein was incubated overnight at 4 °C with beads carrying 200 μg of GST–CoHMGR or GST. After washing and elution, pull-down fractions and 5–10% input samples were analyzed by SDS–PAGE, immunoblotting, and silver staining for subsequent identification of candidate CoHMGR-associated proteins.

### 2.7 SDS–PAGE, immunoblotting and LC–MS/MS analysis of candidate CoHMGR-associated proteins

Pull-down eluates and corresponding input samples were resolved on 4–20% gradient SDS–PAGE gels under reducing conditions. Gels were stained with Coomassie Brilliant Blue or transferred to 0.45 μm polyvinylidene fluoride membranes for immunoblotting. GST and GST–CoHMGR were detected with a mouse anti-GST monoclonal antibody (1:10,000), and His–CoPP1 was detected with a rabbit anti-His polyclonal antibody (1:5,000). Horseradish peroxidase-conjugated secondary antibodies and enhanced chemiluminescence were used for signal detection.

Pull-down eluates from GST and GST–CoHMGR samples at T-0 and T-36 were also subjected to silver staining. Differential bands preferentially recovered in the GST–CoHMGR fractions were excised and digested in-gel with sequencing-grade trypsin. The resulting peptides were analyzed using an UltiMate 3000 RSLCnano system coupled to a Q Exactive HF mass spectrometer (Thermo Scientific).

Raw data were processed in MaxQuant v1.6.6 with the Andromeda search engine against a Camellia protein database downloaded from UniProt on February 4, 2024, supplemented with common contaminants. Trypsin/P specificity with up to two missed cleavages was allowed. Carbamidomethylation of cysteine was set as a fixed modification, whereas methionine oxidation and protein N-terminal acetylation were treated as variable modifications. Peptide- and protein-level false discovery rates were controlled at 1%. Label-free protein abundance was estimated using intensity-based absolute quantification values. Reverse hits, potential contaminants, and proteins identified only by modified sites were excluded. For each GST–CoHMGR versus GST comparison, log_2_-transformed intensity values were analyzed using a two-sided Student’s t-test across replicate pull-down experiments, followed by Benjamini–Hochberg correction. Proteins with a false discovery rate < 0.05 and |log_2_ fold change| ≥ 0.585 were defined as differentially enriched candidate CoHMGR-associated proteins.

### 2.8 Statistical analysis

All physiological and biochemical measurements (including squalene content and HMGR-related ELISA readout) were performed on three independent biological replicates per treatment. Data are presented as means ± standard deviation (SD). Differences among multiple treatments were evaluated by one-way analysis of variance (ANOVA), followed by Tukey’s multiple comparison test when the ANOVA was significant. For comparisons involving only two groups, Student’s *t*-test was used. Data were checked for normality and homogeneity of variance and log-transformed when necessary. A significance level of *p* < 0.05 was adopted throughout. Statistical analyses and graphing were carried out using R and GraphPad Prism.

## 3 Results

### 3.1 Postharvest HTHH treatment coincides with rapid squalene accumulation and changes in an HMGR-related ELISA readout

**Figure 1:**
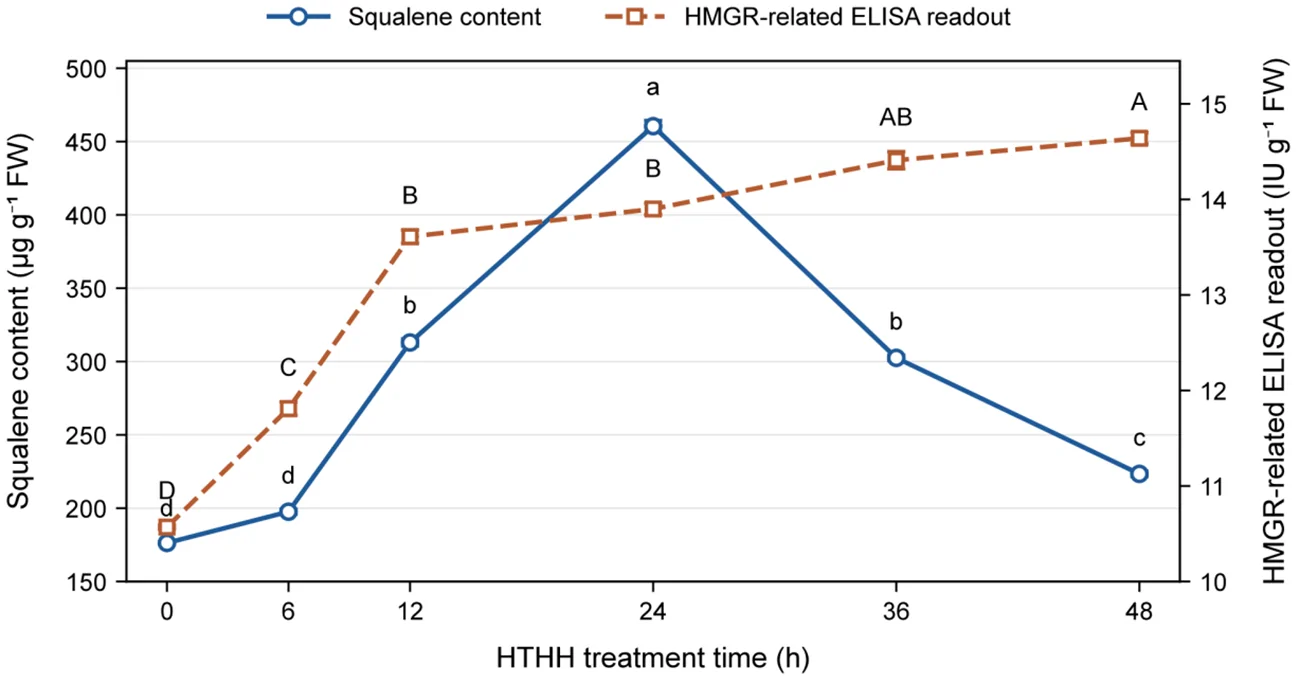
Time-course changes in squalene content and the HMGR-related ELISA readout in postharvest *Camellia oleifera* seeds during HTHH treatment. Squalene content (left y-axis) is expressed as μg g⁻¹ fresh weight (FW), and the HMGR-related ELISA readout (right y-axis) is expressed as IU g⁻¹ FW. Values represent the means ± SD of three biological replicates. Different lowercase letters indicate significant differences in squalene content, and different uppercase letters indicate significant differences in the HMGR-related ELISA readout (one-way ANOVA followed by Tukey’s HSD test, p < 0.05).

To define the early biochemical response of postharvest *C. oleifera* seeds to HTHH treatment, we monitored squalene content and the HMGR-related ELISA readout over a 48 h time course at 35 °C and 95% relative humidity (**Figure 1**). Squalene content remained close to the initial level during the first 6 h, with a value of 177.6 μg g⁻¹ FW at T-0. It then increased markedly by T-12 and reached 461.7 μg g⁻¹ FW at T-24, corresponding to an approximately 2.6-fold increase relative to T-0. Although squalene content declined at T-36 and T-48, it remained above the initial level, indicating that the main accumulation phase occurred within the first 24 h of HTHH exposure. The HMGR-related ELISA readout followed a slower and more sustained trajectory. This parameter increased from 10.7 IU g⁻¹ FW at T-0 to approximately 13.7–14.0 IU g⁻¹ FW by T-24 and reached approximately 14.7 IU g⁻¹ FW at T-48. The two measurements exhibited distinct temporal profiles: squalene content peaked at T-24 and subsequently declined, whereas the HMGR-related ELISA readout remained elevated through T-48. Based on the temporal pattern of squalene accumulation, T-0, T-12, and T-24 were selected for subsequent transcriptomic and metabolomic analyses to represent the baseline, rising, and peak phases, respectively.

### 3.2 Predominant CoHMGR2 transcript abundance and coordinated transcriptional remodeling of the mevalonate pathway during HTHH stress

**Figure 2:**
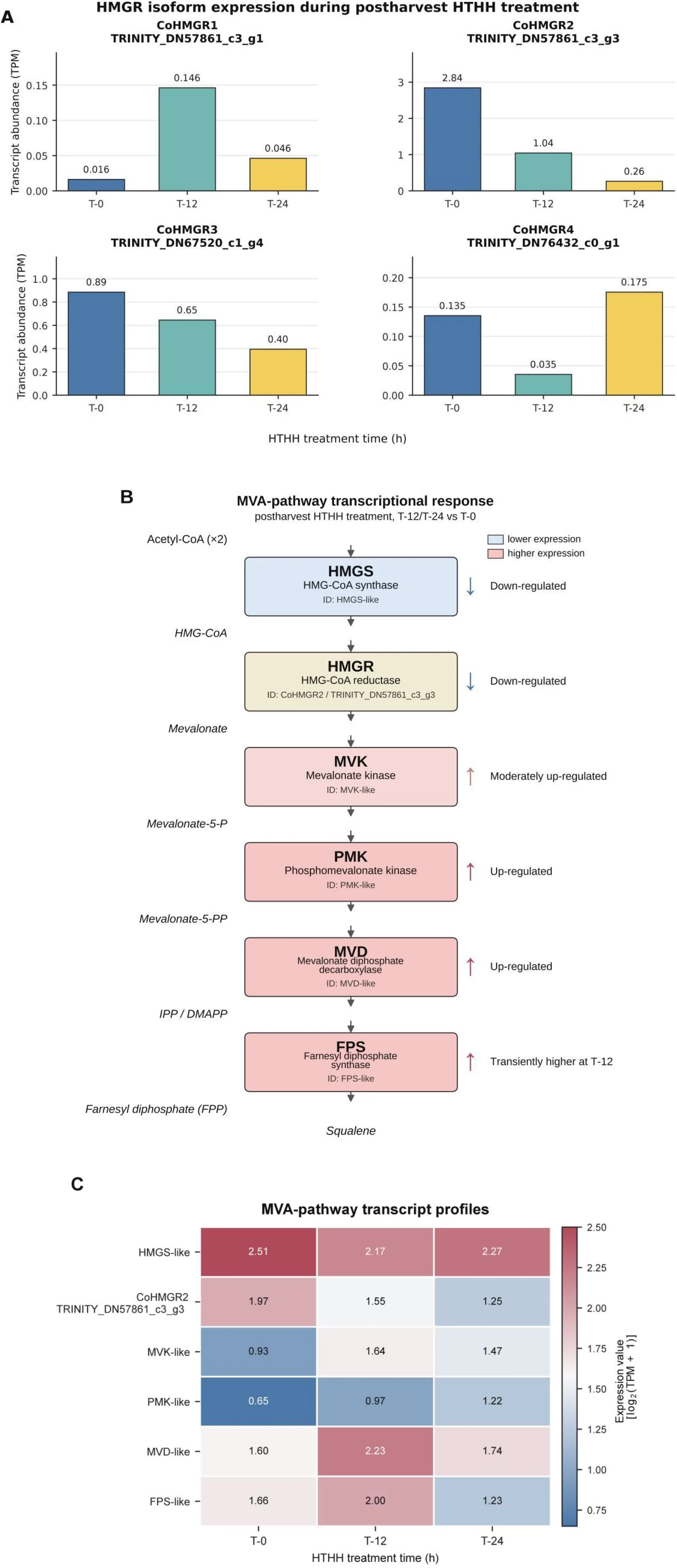
HMGR-family transcript abundance and MVA-pathway transcriptional patterns in Camellia oleifera seeds during postharvest HTHH treatment. **(A)** Transcript abundance of four expressed *CoHMGR* family members at T-0, T-12, and T-24, expressed as transcripts per million (TPM). **(B)** The directional annotations summarize representative transcriptional responses during HTHH treatment; the FPS-like transcript showed a transient increase at T-12 before declining below the T-0 level at T-24. **(C)** Heatmap showing the expression profiles of selected MVA-pathway genes. Colors represent log_2_ (TPM + 1).

To characterize the transcriptional response of the *CoHMGR* gene family to postharvest HTHH treatment, the transcript abundance of four expressed *CoHMGR* family members was examined at T-0, T-12, and T-24 (**Figure 2A**). *CoHMGR1* showed a transient increase at T-12, followed by a decline at T-24. *CoHMGR2* was the most abundant family member at T-0 and T-12, but its transcript abundance decreased markedly during treatment. *CoHMGR3* remained low in abundance and also declined from T-0 to T-24; however, because its decrease was less pronounced than that of *CoHMGR2*, *CoHMGR3* showed a higher transcript abundance than *CoHMGR2* at T-24. *CoHMGR4* remained low in absolute abundance but decreased at T-12 and subsequently recovered at T-24. Collectively, the four CoHMGR family members displayed distinct temporal expression patterns, with *CoHMGR2* predominating at the earlier time points and *CoHMGR3* having the highest relative transcript abundance at T-24.

**Figure 2B** illustrates the representative enzymatic steps of the mevalonate pathway from acetyl-CoA to squalene and provides the pathway framework for the transcript profiles presented in **Figure 2C**. The expression heatmap showed distinct temporal responses among the selected mevalonate-pathway genes (**Figure 2C**). Transcripts encoding 3-hydroxy-3-methylglutaryl-CoA synthase were less abundant at T-12 and T-24 than at T-0. In contrast, mevalonate kinase-like transcripts increased moderately, whereas phosphomevalonate kinase-like and mevalonate diphosphate decarboxylase-like transcripts were more abundant at both T-12 and T-24. Farnesyl diphosphate synthase-like transcript abundance increased transiently at T-12 but declined below the T-0 level at T-24. Together, these results show isoform-specific changes within the HMGR family and distinct temporal responses among downstream mevalonate-pathway genes during HTHH treatment.

### 3.3 Signature metabolite modules characterize the metabolic context of the HTHH-responsive squalene-accumulation phase

**Figure 3:**
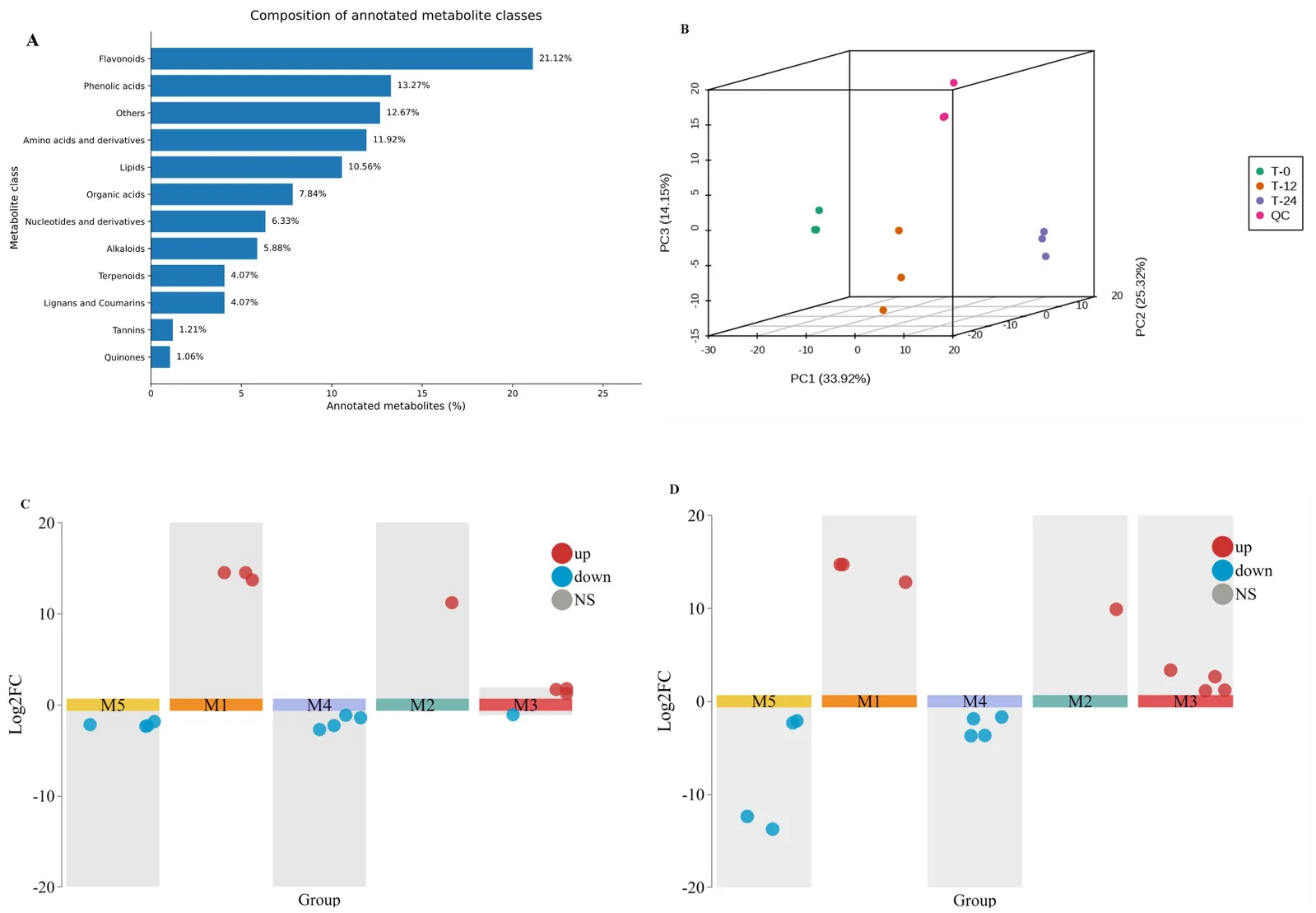
Metabolomic overview and signature metabolite modules in postharvest *Camellia oleifera* seeds under high-temperature and high-humidity (HTHH) treatment. Seeds were sampled at T-0, T-12, and T-24. **(A)** Relative distribution of annotated metabolites among major chemical classes. **(B)** Three-dimensional principal component analysis score plot of the quantified metabolite profiles; QC, quality-control samples. **(C, D)** log_2_ fold-change distributions of selected signature metabolites in the T-0 versus T-12 and T-0 versus T-24 comparisons, respectively. The metabolites were grouped into five modules (M1–M5) according to chemical class, pathway annotation, and temporal response pattern. Red, blue, and gray points indicate significantly increased, significantly decreased, and non-significant metabolites, respectively.

At the metabolome-wide level, the annotated metabolites were distributed across several major chemical classes, including flavonoids, phenolic acids, organic acids, lipids, nucleotides and their derivatives, amino acids and their derivatives, and related categories (**Figure 3A**). Three-dimensional PCA of the quantified metabolite matrix separated T-0, T-12, and T-24 *C. oleifera* seed samples into distinct temporal clusters, whereas quality-control (QC) samples clustered tightly together (**Figure 3B**). This pattern indicated robust analytical reproducibility and a pronounced HTHH-associated metabolic shift. The time-dependent metabolomic separation paralleled the early postharvest window in which squalene accumulation was most pronounced and coincided with changes in the HMGR-related ELISA readout.

To characterize metabolic features associated with this HTHH-responsive squalene-accumulation window, we integrated chemical class, pathway annotation, and time-course response patterns and selected 16 metabolites with large and consistent changes as signature metabolites. These metabolites were organized into five modules, M1–M5 (**Figures 3C, D**). The module-based log_2_ fold-change distributions showed generally concordant patterns across the T-0 versus T-12 and T-0 versus T-24 comparisons, with larger absolute changes observed at T-24 for most signature metabolites (**Figures 3C, D**). Module M5, comprising D-ribose, LysoPC 19:1, adenosine 5′-monophosphate, and inosine 5′-monophosphate, decreased markedly after HTHH treatment, with absolute log_2_ fold changes exceeding 10 for some metabolites. In contrast, the M1 polyols and organic acids, including oxalic acid, D-mannitol, and dulcitol, increased substantially.

The M4 metabolites γ-aminobutyric acid, N-acetylputrescine, pyroglutamic acid, and L-γ-glutamyl-L-leucine decreased in both comparisons. Syringaresinol in M2 and the flavonoid-related metabolites in M3—including phloretin-2′-O-glucoside, 3′-O-methyl-epicatechin, apigenin-6-C-(2″-glucosyl)arabinoside, and apigenin-7-O-(2″-sinapoyl)glucuronide—increased at T-12 and showed larger increases at T-24. Collectively, the 16 signature metabolites showed coordinated decreases in nucleotide- and membrane-lipid-related compounds, accumulation of polyols and organic acids, and time-dependent changes in nitrogen-associated and phenylpropanoid–flavonoid metabolites.

### 3.4 HTHH-induced CoPP1c expression and *in vitro* CoHMGR–CoPP1 association

**Figure 4:**
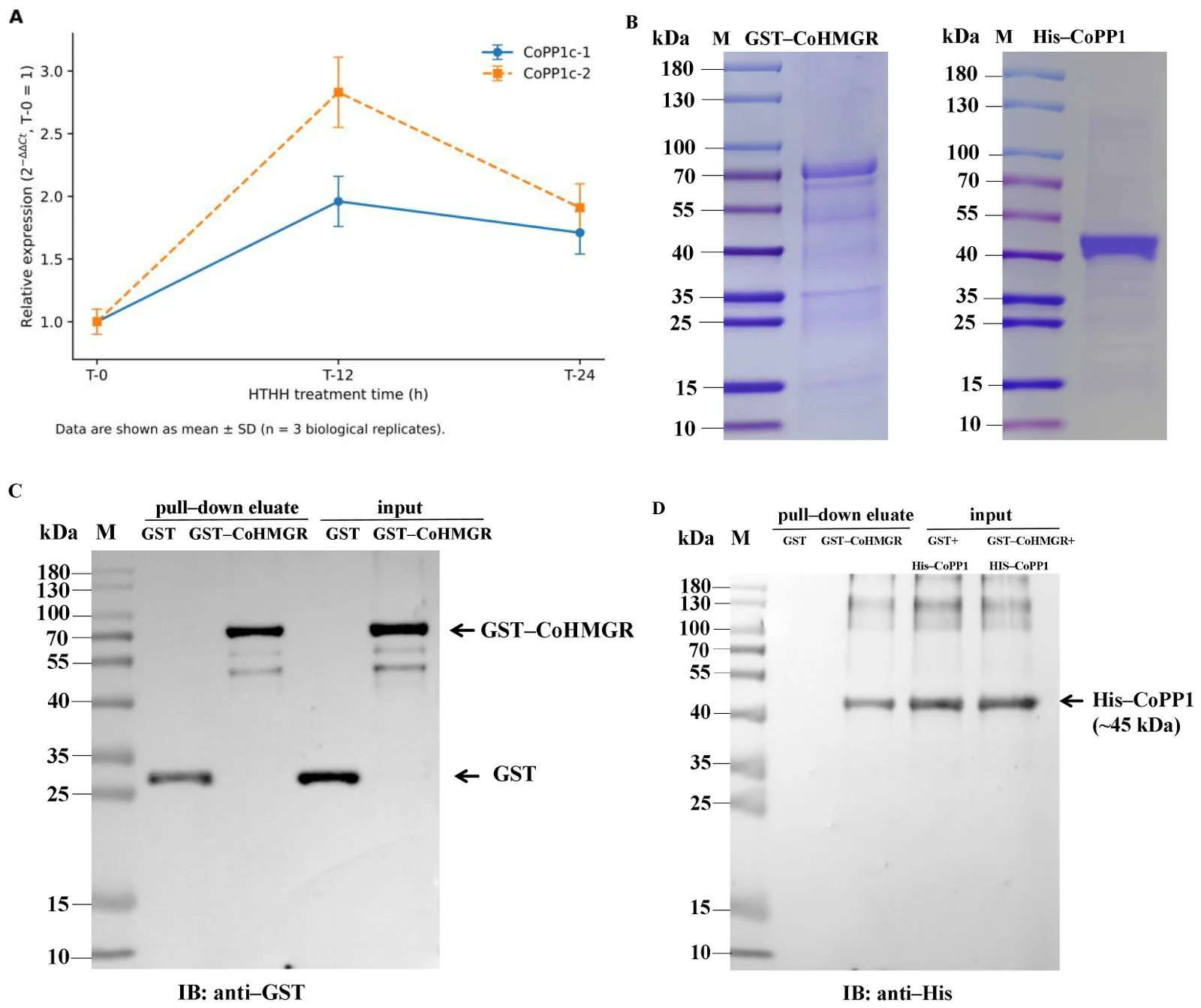
HTHH-induced CoPP1c expression and *in vitro* association between recombinant CoHMGR and CoPP1. **(A)** Reverse transcription quantitative PCR analysis of *CoPP1c-1* and *CoPP1c-2* expression in postharvest *Camellia oleifera* seeds at T-0, T-12, and T-24 under high-temperature and high-humidity treatment. Relative transcript levels were calculated using the 2^−ΔΔCt^ method with T-0 as the calibrator. Values are means ± SD of three biological replicates. **(B)** Coomassie Brilliant Blue-stained SDS–PAGE of purified GST–CoHMGR and His–CoPP1. Expected molecular masses were approximately 78 and 45 kDa, respectively. M, molecular-mass marker. **(C)** Anti-GST immunoblot of input and pull-down fractions showing GST–CoHMGR and GST bait recovery. **(D)** Anti-His immunoblot of input and pull-down fractions. His–CoPP1 was detected in the GST–CoHMGR eluate but not in the GST-only control.

HTHH treatment not only coincided with increased HMGR-related ELISA readout and squalene accumulation (Sections 3.1–3.3), but was also accompanied by marked changes in the expression of PP1 catalytic-subunit genes in *C. oleifera* seeds. RT-qPCR analysis showed that both *CoPP1c-1* and *CoPP1c-2* were induced during HTHH treatment (**Figure 4A**). At T-12, *CoPP1c-1* and *CoPP1c-2* transcript levels increased approximately 2.0- and 2.8-fold, respectively, relative to T-0 and remained approximately 1.7- to 1.9-fold relative to T-0 at T-24. These HTHH-responsive transcriptional changes suggested that PP1-related components may be associated with the broader response context accompanying HMGR-related changes and squalene accumulation, although the expression data alone do not establish regulatory involvement. To examine this possibility at the protein level, we next performed recombinant-protein pull-down assays to test whether a heat-responsive CoHMGR isoform could associate with a PP1 catalytic subunit *in vitro*. Coomassie-stained SDS–PAGE showed predominant bands near the expected molecular masses of approximately 78 and 45 kDa for GST–CoHMGR and His–CoPP1, respectively (**Figure 4B**), indicating sufficient purity for pull-down assays.

In the GST pull-down experiment, GST–CoHMGR or GST alone was immobilized on glutathione beads and incubated with excess His–CoPP1. Anti-GST immunoblotting confirmed recovery of GST–CoHMGR and GST in their respective pull-down eluates (**Figure 4C**). When the same membranes were probed with anti-His antibody, a strong His–CoPP1 signal was detected in the eluates from the GST–CoHMGR pull-down, but no corresponding band was observed in the GST control (**Figure 4D**). Taken together, these results support an *in vitro* association between recombinant CoHMGR and the PP1 catalytic subunit under the assay conditions used.

### 3.5 Pull-down enrichment and KEGG annotation of candidate CoHMGR-associated proteins during HTHH treatment

**Figure 5:**
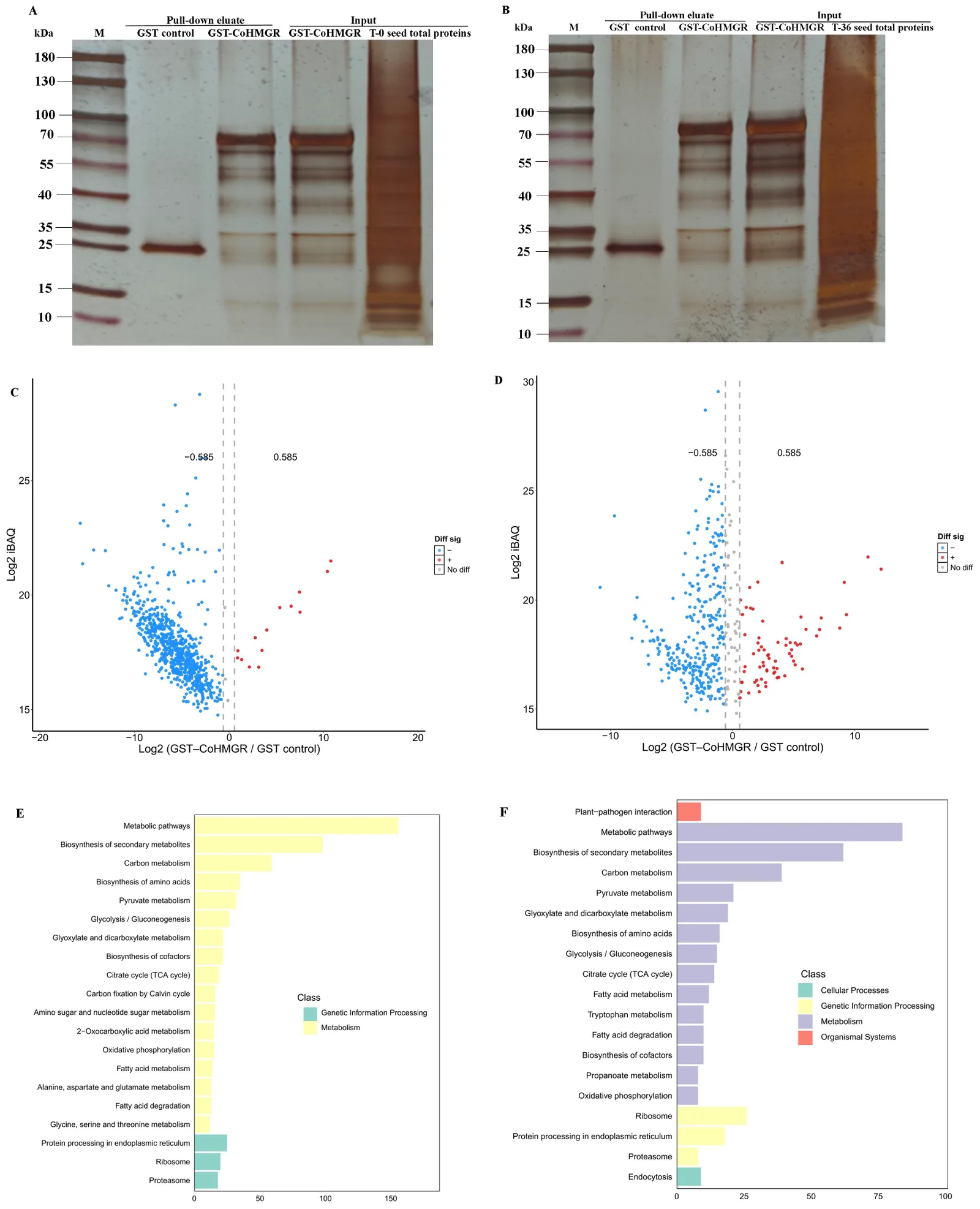
GST–CoHMGR pull-down profiling of candidate CoHMGR-associated proteins during HTHH treatment. **(A, B)** Silver-stained SDS–PAGE profiles of proteins recovered with GST or GST–CoHMGR from *Camellia oleifera* seed extracts at T-0 **(A)** and T-36 **(B)**. Because the two time points were analyzed on separate gels, comparisons are qualitative within each time point. **(C, D)** Differential enrichment–abundance plots for GST–CoHMGR versus GST at T-0 **(C)** and T-36 **(D)**. The x-axis shows log_2_ (GST–CoHMGR/GST), and the y-axis shows log_2_-transformed iBAQ abundance. Colored points indicate proteins meeting the enrichment criteria of |log_2_FC| ≥ 0.585 and statistical significance; gray points indicate non-significant proteins. **(E, F)** KEGG pathway annotations of differentially enriched candidate proteins at T-0 **(E)** and T-36 **(F)**. The x-axis indicates the number of annotated proteins, and bar colors denote higher-level KEGG categories. The T-36 dataset contained more enriched proteins and broader functional representation than the T-0 dataset.

To profile candidate CoHMGR-associated proteins during HTHH treatment, GST–CoHMGR pull-down assays were performed using total-protein extracts from *C. oleifera* seeds collected at T-0 and T-36. Silver-stained SDS–PAGE gels showed differential banding patterns between GST–CoHMGR pull-down samples and the GST control at both time points (**Figures 5A, B**), supporting the presence of candidate CoHMGR-associated proteins for subsequent liquid chromatography–tandem mass spectrometry (LC–MS/MS) analysis. Because the T-0 and T-36 samples were resolved on separate gels, these silver-stained patterns were interpreted qualitatively and were not used for direct between-time-point comparisons of band intensity or protein abundance.

Based on quantitative proteomics, differential enrichment-abundance plots were generated for the GST–CoHMGR pull-down datasets collected at T-0 and T-36 (**Figures 5C, D**). In this study, proteins with |log_2_FC| ≥ 0.585, corresponding to an approximately 1.5-fold difference, together with statistical significance, were defined as differentially enriched candidate CoHMGR-associated proteins. At T-0, only a limited subset of proteins exceeded this threshold. By contrast, a larger number of proteins met the same criteria at T-36, indicating that prolonged HTHH treatment was associated with a broader GST–CoHMGR pull-down-enriched protein set. Because these data were derived from affinity pull-down followed by LC–MS/MS, they are interpreted here as differential enrichment patterns within candidate CoHMGR-associated proteins, rather than as direct evidence of specific endogenous interactions or functional recruitment events.

Some enriched proteins may reflect nonspecific binding, high cellular abundance, or indirect association with the bait.

KEGG enrichment analysis of the differentially enriched candidate CoHMGR-associated proteins showed that, at T-0, the enriched annotations were dominated by central metabolic pathways, including “Metabolic pathways,” “Biosynthesis of secondary metabolites,” “Carbon metabolism,” “Glycolysis/Gluconeogenesis,” and “Citrate cycle (TCA cycle)” (**Figure 5E**). At T-36, these metabolic annotations remained prominent, while additional categories such as “Plant–pathogen interaction,” “Protein processing in endoplasmic reticulum,” “Ribosome,” “Fatty acid metabolism,” and “Endocytosis” were also represented among the enriched proteins (**Figure 5F**). Collectively, these results indicate that prolonged HTHH treatment was associated with a broader functional annotation profile in the GST–CoHMGR pull-down dataset. This pattern provides an annotation-based proteomic context for CoHMGR-associated responses during postharvest HTHH treatment, but does not by itself establish direct regulatory coupling or interaction specificity.

### 3.6 CoHMGR pull-down profiling reveals functional protein contexts enriched after 36 h of HTHH treatment

**Figure 6:**
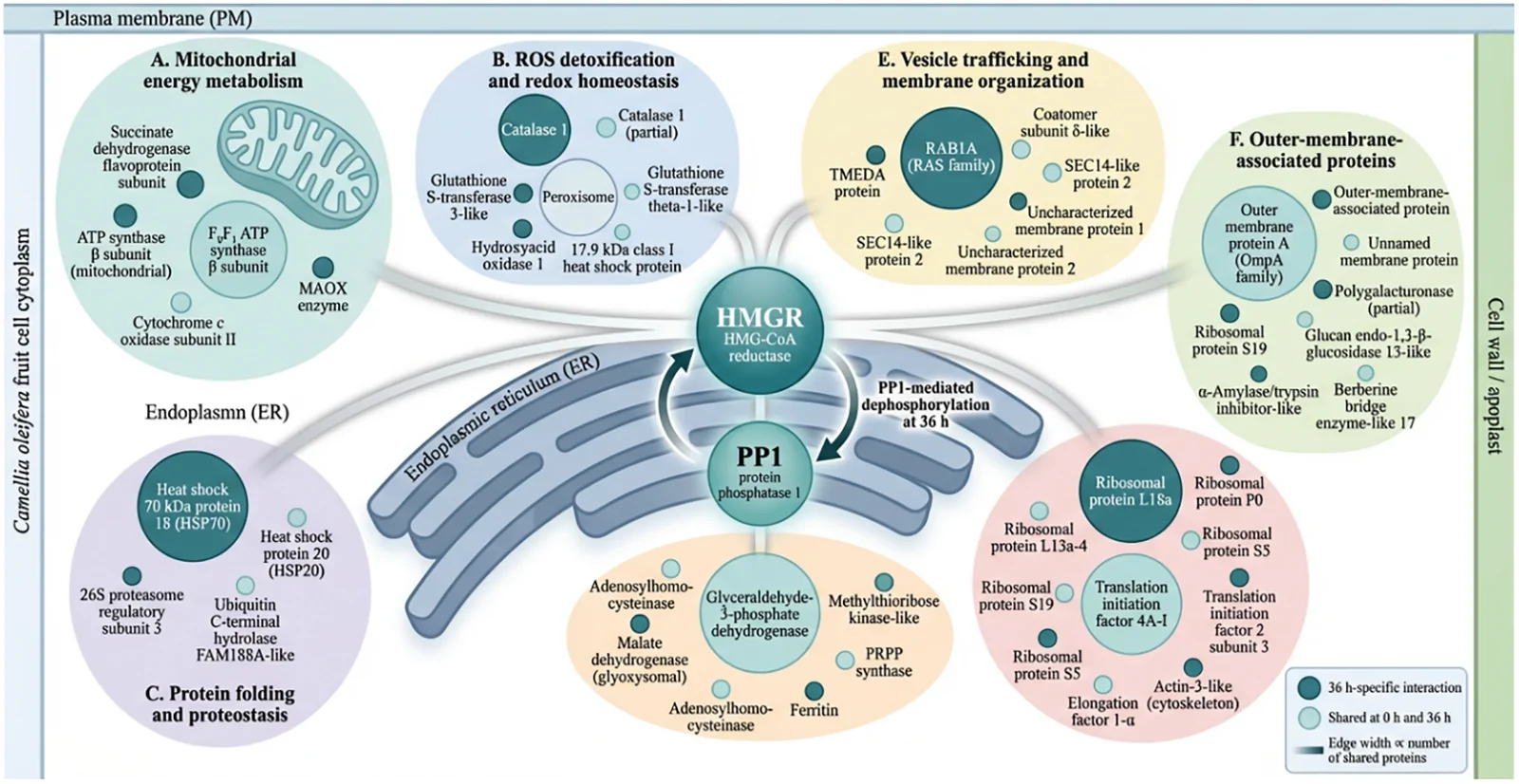
Functional annotation overview of candidate CoHMGR-associated proteins enriched after 36 h of high-temperature and high-humidity treatment. Candidate proteins identified from the GST–CoHMGR pull-down dataset were classified into seven functional categories: **(A)** mitochondrial energy metabolism; **(B)** reactive oxygen species detoxification and redox homeostasis; **(C)** protein folding and proteostasis; **(D)** central carbon and methyl metabolism; **(E)** vesicle trafficking and membrane organization; **(F)** outer membrane- or apoplast-associated proteins; and **(G)** translation- and ribosome-related proteins. CoHMGR is positioned near the endoplasmic reticulum membrane to represent its membrane-associated localization. Dark nodes indicate proteins detected only at 36 h, whereas light nodes indicate proteins detected at both 0 and 36 h. Node size represents relative enrichment in the GST–CoHMGR pull-down dataset, and edge thickness indicates the number of proteins assigned to each category. The schematic summarizes functional annotations of candidate proteins and does not imply validated endogenous interactions or direct regulatory relationships.

To explore the protein context associated with CoHMGR during prolonged HTHH exposure, we performed GST–CoHMGR pull-down followed by LC–MS/MS using total-protein extracts from *C. oleifera* seeds collected at T-0 and T-36. Under the defined enrichment criteria, 14 candidate CoHMGR-associated proteins were detected at T-0, whereas 69 proteins met the same criteria at T-36 (**Figure 6**). This increase in pull-down-enriched proteins suggested that the CoHMGR-associated protein context became broader after 36 h of HTHH treatment. Because the pull-down assay was based on affinity enrichment from seed protein extracts and does not by itself establish endogenous complex formation or functional specificity, these proteins are referred to here as candidate CoHMGR-associated proteins.

To summarize the major functional patterns within the T-36 pull-down dataset, the enriched proteins were grouped into seven annotation-based categories using predicted subcellular localization and KEGG/GO information: mitochondrial energy metabolism, ROS detoxification and redox homeostasis, protein folding and proteostasis, central carbon and methyl metabolism, vesicle trafficking and membrane organization, outer-membrane- or apoplast-associated proteins, and translation/ribosome-related proteins (**Figure 6**). These categories should be interpreted as functional annotations of the pull-down-enriched protein set rather than as experimentally validated interaction modules.

The mitochondrial-energy category included proteins annotated as succinate dehydrogenase flavoprotein subunit, F-type ATP synthase β subunit, and cytochrome c oxidase subunit II. Their enrichment suggests that proteins associated with respiratory energy metabolism were represented in the CoHMGR pull-down dataset at T-36. The ROS/redox category included catalase 1, glutathione S-transferase 3-like, glutathione S-transferase theta-1-like, and hydroxyacid oxidase 1, indicating that redox-related proteins were also present among the T-36-enriched candidates. The protein-folding/proteostasis category contained heat shock 70 kDa protein 18, heat shock protein 20, 26S proteasome regulatory subunit 3, and ubiquitin C-terminal hydrolase FAM188A-like, suggesting that protein-quality-control-related annotations were represented in the same dataset.

The central carbon and methyl-metabolism category included glyceraldehyde-3-phosphate dehydrogenase, malate dehydrogenase, adenosylhomocysteinase, methylthioribose kinase-like protein, and phosphoribosyl pyrophosphate (PRPP) synthase. These annotations indicate that carbon- and methyl-metabolism-related proteins were captured in the CoHMGR pull-down fraction. Vesicle-trafficking and membrane-organization proteins, including RAB1A, TMEDA protein, SEC14-like protein 2, and several uncharacterized membrane-associated proteins, were also detected. Outer-membrane- or apoplast-associated annotations formed a separate group, although their biological origin and relevance require further validation. Finally, ribosomal proteins and translation factors were enriched among the T-36 candidates, suggesting that translation-related proteins were also represented in the pull-down-enriched protein set.

**Figure 6** summarizes the functional annotations of candidate proteins enriched in the GST–CoHMGR pull-down dataset after 36 h of HTHH treatment. The candidates were assigned to seven functional categories: mitochondrial energy metabolism, redox homeostasis, protein folding and proteostasis, central carbon and methyl metabolism, vesicle trafficking and membrane organization, outer membrane- or apoplast-associated processes, and translation- and ribosome-related functions. Proteins detected only at T-36 and those shared between the T-0 and T-36 datasets are distinguished in the schematic. The T-36-specific candidates included proteins associated with respiratory metabolism, antioxidant defense, molecular chaperone activity, protein turnover, and membrane trafficking.

## 4 Discussion

### 4.1 PP1-associated HMGR responses during HTHH stress

The time-resolved biochemical and transcriptomic data revealed a clear temporal disconnect among squalene accumulation, HMGR-related immunoreactivity, and HMGR-family transcription during postharvest HTHH treatment. Squalene remained close to its initial level during the first 6 h, increased sharply between 12 and 24 h, and then declined while remaining above baseline through 48 h. By contrast, the HMGR-related ELISA readout increased more gradually and remained elevated from 24 to 48 h. At the transcript level, *CoHMGR2* was the predominant HMGR family member at T-0 and T-12, but its abundance decreased progressively, whereas *CoHMGR3* became the most abundant detected HMGR-family transcript at T-24. Several downstream mevalonate-pathway genes showed treatment-responsive expression, with PMK-like and MVD-like transcripts remaining elevated at both T-12 and T-24, whereas FPS-like transcript abundance increased only transiently at T-12. These contrasting patterns indicate that the increase in squalene was not driven by coordinated transcriptional induction of the predominant HMGR transcript alone, but occurred together with altered HMGR-family transcript composition and increased expression of downstream pathway components. This interpretation is consistent with the central rate-limiting position of HMGR in the mevalonate pathway, while also indicating that pathway output cannot be inferred from HMGR transcript abundance alone (Stermer et al., 1994). The sustained HMGR-related ELISA signal should nevertheless be interpreted cautiously. The assay quantifies antibody-recognized HMGR-related material rather than the catalytic conversion of HMG-CoA to mevalonate. Variation in the signal may therefore reflect changes in protein abundance, isoform cross-reactivity, epitope accessibility, protein conformation, or degradation state. The assay cannot distinguish catalytically active from inactive HMGR or resolve phosphorylation-dependent changes in catalytic efficiency. Accordingly, the increased ELISA readout during HTHH indicates greater HMGR-related immunoreactivity, but does not establish enhanced HMGR activity or PP1-mediated activation. The temporal divergence between the transient squalene peak and the more sustained immunoreactive signal further suggests that this readout alone does not account for pathway output. Similar uncoupling between HMGR transcription and protein-level regulation has been reported in *Arabidopsis thaliana*, where HMGR abundance and activity are strongly influenced by phosphorylation and proteolysis (Dale et al., 1995; Leivar et al., 2011). Direct measurements of HMGR catalytic activity, protein abundance, and phosphorylation state will therefore be required to determine whether post-transcriptional or post-translational regulation contributes to the HTHH response.

The metabolomic data further place the squalene-accumulation phase within a broader reorganization of seed metabolism. The depletion of D-ribose, adenosine 5′-monophosphate, inosine 5′-monophosphate, and LysoPC 19:1 indicates marked changes in nucleotide-related and membrane-lipid-associated metabolite pools. In contrast, the accumulation of oxalic acid, D-mannitol, and dulcitol is consistent with altered carbon allocation and osmotic adjustment under hot–humid conditions. Concurrent decreases in γ-aminobutyric acid-, polyamine-, and γ-glutamyl-related metabolites, together with increases in syringaresinol and several flavonoids, further indicate coordinated changes in nitrogen metabolism, redox balance, and phenylpropanoid metabolism. Although these metabolites are not direct products of the mevalonate pathway, their coordinated responses show that squalene accumulation occurred within a wider stress-associated metabolic transition rather than as an isolated pathway event. These observations provide metabolic context for the HMGR- and PP1-associated responses but do not establish direct regulation of individual metabolites by either protein. Within this broader response, *CoPP1c-1* and *CoPP1c-2* were most strongly induced at T-12, immediately before the squalene maximum at T-24. In parallel, recombinant-protein pull-down assays showed that CoHMGR and CoPP1 can associate under the defined *in vitro* conditions. The temporal induction of the PP1 catalytic-subunit genes, together with this biochemical association, supports placement of PP1 within a candidate CoHMGR-associated regulatory context during the squalene-accumulation phase. However, gene induction does not demonstrate substrate recognition, and the pull-down assay does not establish interaction under native cellular conditions, direct dephosphorylation of CoHMGR, or a change in its catalytic activity. The present evidence therefore supports a testable PP1-linked regulatory hypothesis rather than a resolved phosphatase mechanism.

This candidate PP1-associated context should be considered alongside, rather than in opposition to, the established PP2A-centered framework of plant HMGR regulation. In *A. thaliana*, PP2A holoenzymes containing specific B-type regulatory subunits influence HMGR transcript abundance, protein stability, and enzymatic activity under environmental stress (Leivar et al., 2011). PP1, by contrast, functions through diverse regulatory and targeting proteins that can direct the catalytic subunit to distinct substrates and subcellular compartments, allowing it to attenuate, sustain, or reset signaling outputs in a context-dependent manner (Ceulemans and Bollen, 2004; Virshup and Shenolikar, 2009). Our findings raise the possibility that PP1 represents an additional regulatory component associated with HMGR responses in a woody oil crop under prolonged postharvest HTHH treatment. However, the direction and functional consequence of this association remain unresolved. A cautious working model is therefore that PP1-related responses form one layer of a broader regulatory network involving HMGR-family transcript redistribution, induction of downstream mevalonate-pathway genes, and extensive metabolic adjustment. Identification of the PP1 regulatory subunits associated with CoHMGR, mapping of candidate docking and phosphorylation sites, and direct phosphatase and enzyme-activity assays will be required to determine how this putative module is integrated into the wider stress-signaling network (Cohen, 2002; Ceulemans and Bollen, 2004; Virshup and Shenolikar, 2009).

### 4.2 Functional context and validation priorities for candidate CoHMGR-associated proteins

Protein phosphatase 1 functions as a modular serine/threonine phosphatase whose substrate specificity is conferred largely by a diverse repertoire of protein phosphatase 1-interacting proteins. These partners can act as targeting subunits, scaffolds, substrates, or inhibitors, thereby directing individual holoenzymes to defined substrates and subcellular compartments (Ceulemans and Bollen, 2004; Virshup and Shenolikar, 2009; Bollen et al., 2010). Although plant protein phosphatase 1 has been implicated in metabolic, hormonal, light-responsive, and stress-signaling processes, its connection to the mevalonate pathway remains poorly defined. Against this background, the GST–CoHMGR pull-down proteome provides a hypothesis-generating view of the protein environment recovered with CoHMGR during prolonged HTHH exposure. Its principal value lies not in defining a validated interactome, but in identifying functional classes and individual candidates for subsequent mechanistic testing.

The marked increase in differentially enriched proteins from T-0 to T-36 indicates that the protein context recovered with GST–CoHMGR became broader during prolonged treatment. At T-36, candidate proteins were assigned to mitochondrial energy metabolism, redox homeostasis, protein folding and proteostasis, central carbon and methyl metabolism, vesicle trafficking and membrane organization, and translation-related processes. The representation of respiratory-chain and central-carbon-metabolism proteins is compatible with the substantial ATP and reducing-power requirements of the mevalonate pathway and downstream sterol and triterpene biosynthesis. Similarly, the recovery of catalase, glutathione transferases, heat-shock proteins, proteasome-associated components, and ubiquitin-related factors indicates that redox control and protein quality control were prominent features of the late HTHH pull-down dataset. These annotations are biologically compatible with the metabolic and proteostatic demands of prolonged hot–humid exposure, but they do not demonstrate that the recovered proteins directly regulate CoHMGR.

Vesicle-trafficking and membrane-organization proteins may be particularly relevant because plant HMGR is an endoplasmic-reticulum-associated membrane protein whose abundance, localization, and turnover can be influenced by its membrane environment. RAB1A, TMEDA, and SEC14-like protein 2 therefore represent plausible candidates for examining whether membrane trafficking or lipid-transfer processes contribute to the subcellular organization of CoHMGR. Selected chaperones and proteostasis-related proteins also warrant further analysis because they could influence CoHMGR folding, stability, or degradation. By contrast, highly abundant central metabolic enzymes, ribosomal proteins, and translation factors should initially be interpreted more cautiously, as their recovery may reflect cellular abundance, indirect association, or broadly adhesive behavior rather than specific recognition of the bait. Affinity pull-down data inherently capture a mixture of direct binders, members of larger protein assemblies, transiently associated proteins, and nonspecific background. Candidate prioritization should therefore integrate enrichment magnitude, statistical significance, reproducibility across biological replicates, preferential recovery at T-36, predicted subcellular compatibility with membrane-associated CoHMGR, and functional relevance to HMGR regulation. On these criteria, the vesicle-trafficking, membrane-organization, and selected proteostasis candidates provide a more focused starting point for validation than broadly abundant metabolic or translation-related proteins. Reciprocal pull-down and co-immunoprecipitation will be required to determine whether these candidates associate specifically with CoHMGR in native extracts, whereas bimolecular fluorescence complementation or related cellular assays can test whether the associations occur in compatible subcellular compartments.

The proposed CoHMGR–CoPP1 relationship likewise remains provisional. The recombinant-protein pull-down establishes that the two proteins can associate under the defined *in vitro* conditions, but does not establish endogenous complex formation, direct substrate recognition, or phosphatase-dependent regulation. Co-immunoprecipitation and bimolecular fluorescence complementation would provide complementary evidence for interaction under native or cellular conditions, while targeted phosphatase assays should determine whether CoPP1 changes the phosphorylation state of CoHMGR. These experiments should be coupled with direct HMGR activity measurements and phosphosite analysis to establish whether any change in phosphorylation alters catalytic function. Identification of the PP1 regulatory or targeting proteins that recruit CoPP1 to CoHMGR will also be essential, because the catalytic subunit alone does not explain substrate specificity within the modular PP1 holoenzyme system (Virshup and Shenolikar, 2009; Bollen et al., 2010). Collectively, the pull-down proteome places CoHMGR within a broader late-stage protein context involving energy metabolism, redox homeostasis, proteostasis, and membrane organization. This framework does not establish a unified signaling complex or direct functional coupling among all recovered proteins. Instead, it defines a prioritized set of experimentally testable associations and extends the PP1-related hypothesis developed in Section 4.1 from a single recombinant-protein association to a broader, but still provisional, cellular context. Validation of specific endogenous interactions, PP1-dependent dephosphorylation, and consequent changes in HMGR catalytic activity will be required before a mechanistic CoPP1–CoHMGR regulatory module can be established.

## 5 Conclusions and prospects

Postharvest treatment at 35 °C and 95% relative humidity induced a rapid but transient increase in squalene accumulation in Camellia oleifera seeds. The distinct temporal patterns of squalene content, the HMGR-related ELISA readout, HMGR-family transcript abundance, and downstream mevalonate-pathway gene expression indicate that this response cannot be explained by transcriptional regulation of the predominant HMGR isoform alone. The induction of two PP1 catalytic-subunit genes, together with the *in vitro* association between recombinant CoHMGR and CoPP1, identifies PP1 as a candidate component of an HMGR-associated regulatory context during the principal squalene-accumulation window. Metabolomic and pull-down proteomic analyses further place this response within broader changes in primary metabolism, redox homeostasis, proteostasis, and membrane organization. However, the present evidence does not establish increased HMGR catalytic activity, endogenous CoHMGR–CoPP1 interaction, or direct PP1-mediated dephosphorylation of CoHMGR. Overall, these findings extend the current PP2A-centered framework of plant HMGR regulation and provide a testable basis for investigating phosphatase-associated control of the mevalonate pathway during postharvest squalene accumulation in a woody oil crop.

## Data Availability

The RNA-seq datasets generated in this study have been deposited in the NCBI Sequence Read Archive under BioProject accession PRJNA1355314. All other data supporting the conclusions of this article are included in the article and its Supplementary Material.
